# Sox6 Directly Silences Epsilon Globin Expression in Definitive Erythropoiesis

**DOI:** 10.1371/journal.pgen.0020014

**Published:** 2006-02-03

**Authors:** Zanhua Yi, Orit Cohen-Barak, Nobuko Hagiwara, Paul D Kingsley, Deborah A Fuchs, Drew T Erickson, Elliot M Epner, James Palis, Murray H Brilliant

**Affiliations:** 1 Department of Pediatrics, University of Arizona, College of Medicine, Tucson, Arizona, United States of America; 2 Department of Internal Medicine, Division of Cardiovascular Medicine, University of California Davis, Davis, California, United States of America; 3 Department of Pediatrics, Center for Pediatric Biomedical Research, University of Rochester Medical Center, Rochester, New York, United States of America; 4 Department of Pathology, University of Arizona, College of Medicine, Tucson, Arizona, United States of America; 5 Department of Hematology & Oncology, Oregon Health & Science University, Portland, Oregon, United States of America; Stanford University, United States of America

## Abstract

Sox6 is a member of the Sox transcription factor family that is defined by the conserved high mobility group (HMG) DNA binding domain, first described in the testis determining gene, *Sry*. Previous studies have suggested that Sox6 plays a role in the development of the central nervous system, cartilage, and muscle. In the Sox6-deficient mouse, *p^100H^,* ɛy globin is persistently expressed, and increased numbers of nucleated red cells are present in the fetal circulation. Transfection assays in GM979 (erythroleukemic) cells define a 36–base pair region of the ɛy proximal promoter that is critical for Sox6 mediated repression. Electrophoretic mobility shift assay (EMSA) and chromatin immunoprecipitation (ChIP) assays demonstrate that Sox6 acts as a repressor by directly binding to the ɛy promoter. The normal expression of Sox6 in wild-type fetal liver and the ectopic expression of ɛy in *p^100H^* homozygous fetal liver demonstrate that Sox6 functions in definitive erythropoiesis. The present study shows that Sox6 is required for silencing of ɛy globin in definitive erythropoiesis and suggests a role for Sox6 in erythroid cell maturation. Thus, Sox6 regulation of ɛy globin might provide a novel therapeutical target in the treatment of hemoglobinopathies such as sickle cell anemia and thalassemia.

## Synopsis

Beta-globin gene switching—the transition from embryonic to fetal to adult synthesis of specific globin chains—results in hemoglobins with different affinity for oxygen. This system is a longstanding paradigm for developmental biology and is directly relevant to human disease, since small amounts of normal embryonic or fetal beta-globins can “balance” the detrimental effect of abnormal or missing adult globins in diseases such as sickle cell anemia and beta-thalassemia.

In the current study, the transcription factor Sox6 was identified as a novel and crucial silencing factor of epsilon (embryonic) globin through a somewhat serendipitous pathway. The authors had previously identified a chromosomal inversion, *p^100H^,* by virtue of its effect on the pink-eyed dilution gene and found that the same inversion also disrupts the *Sox6* gene. Using *p^100H^* mutant mice as a tool for identifying downstream targets of Sox6, the authors discovered that epsilon-globin levels were dramatically elevated, paving the way for a series of molecular genetic experiments demonstrating that Sox6 directly binds to and normally inhibits transcription from the epsilon-globin gene promoter. This work provides fundamental new insights into regulation of globin gene transcription during development, and provides new clues for manipulating globin gene transcription as an approach to treat human blood diseases.

## Introduction

Sry type HMG box (Sox6) is a member of the Sox transcription factor family characterized by the conserved high mobility group (HMG) domain, consisting of 79 amino acids involved in DNA recognition and binding [1]. Sox transcription factors bind to the minor groove of DNA and cause a 70°–85° bend of the DNA that leads to local conformational changes [2,3], while most other transcription factors target the major groove of DNA [4]. Therefore, Sox proteins may perform part of their function as architectural proteins by organizing local chromatin structure and assembling other DNA-bound transcription factors into biologically active, sterically defined multiprotein complexes. Sox6 has been reported to be able to act as either an activator or a repressor, depending on its interactors and its target promoter context [5,6]. Intriguingly, Sox6 has also been shown to act as a general splicing factor that participates in pre-mRNA splicing [7]. Depletion of Sox6 in HeLa cell extracts blocked splicing of multiple substrates, and expression of the HMG domain of either Sox6, Sox9, or Sry in the extracts restored splicing, indicating functional overlap of these proteins [7]. Regardless of how Sox6 functions in regulating gene expression, previous studies have demonstrated that Sox6 is an important regulatory molecule that plays a role in the development of the central nervous system [8–11], cartilage [6,12,13], and muscle [14,15]. A Sox6-null mutant mouse *(p^100H^)* has previously been identified in our laboratory [14]. Mice homozygous for *p^100H^* show delayed growth, develop myopathy and arterioventricular heart block, and die within 2 wk after birth [14]. The *p^100H^* mutant allele is associated with a Chromosome 7 inversion that disrupts both the *p* gene and the *Sox6* gene (and no other gene within 50,000 nucleotides of the chromosomal breakpoints) [14]. Because the *p* gene functions solely in pigmentation [16], the Sox6 transcription factor is implicated in all other phenotypes.

Among the HMG box proteins distantly related to Sry (the first member identified of the Sox transcription factor family) that similarly bind to the minor groove and bend DNA, but without sequence specificity, are the ubiquitously expressed HMG1 and HMG2 proteins [17]. Modulation of DNA structure by these and other HMG proteins can mediate long-range enhancer function on both DNA and chromatin-assembled genes by bringing together distant regions of DNA and associated factors. Specifically, HMG proteins have been shown to modulate β-globin genes [18–21].

The mouse β-globin genes {ɛy, βh1, β-major, and β-minor} are clustered on Chromosome 7 and they are highly homologous to their human counterparts in organizational structure and function [22]. High-level expression of these genes requires a regulatory element, the locus control region that is characterized by a set of nuclease hypersensitive sites spread over 25 kb located 5′ of the ɛy gene [23]. The β-globin genes are expressed in a tissue- and development-specific fashion. In mice, erythropoiesis originates in the embryonic yolk sac where primitive erythroid cells express ɛy and βh-1 globins [22]. At 11.5 d post coitus (dpc), erythropoiesis shifts to the fetal liver where definitive erythroid cells express adult β globins (β major and minor) [22]. The ɛy gene is silenced in definitive erythroid cells. The mechanism of silencing of its human counterpart, ɛ globin, has been studied extensively. In definitive erythropoiesis, ɛ is activated and silenced autonomously [24,25], although in primitive erythropoiesis ɛ also appears to be regulated competitively [26]. The γ-globin to adult β-globin switch is controlled by promoter competition for the LCR [24,25].

All the elements responsible for silencing the ɛ globin gene are within the ɛ gene or in adjacent sequences [27], suggesting that silencing is primarily gene autonomous. Using promoter deletion analyses in transgenic mouse models and cell transfection assays, multiple DNA elements important to the silencing process have been previously identified in both the proximal and the distal ɛ gene promoter [27]. Their corresponding transcription factors, such as GATA-1, YY-1, COUP-TF, and DRED have been identified and shown to directly bind to these DNA elements (as part of protein complexes) to regulate ɛ silencing [27]. Thus, it appears that the silencing of the ɛ gene involves a complicated network of multiple *cis* elements and transacting proteins.

In addition to playing an important role in the development of the central nervous system [8–11], cartilage [6,12,13], and muscle [14,15], it was shown that Sox6 is upregulated in long-term hematopoiesis stem cells (LT-HSC) compared with multipotent progenitors of adult mouse bone marrow lineage [28]. In this study, we describe that Sox6 also exerts pleiotropic effects on erythropoiesis. These effects include delayed maturation of erythrocytes (that normally enucleate prior to entering the bloodstream [27]) and higher expression of embryonic globin genes. The most extreme effect is the persistence of high expression of the embryonic ɛy globin gene. Here we describe and characterize the effects of Sox6 on the ɛy globin gene. We show that Sox6 binds to the proximal promoter of ɛy globin and represses its transcription**.** In wild-type (WT) mice, Sox6 is not expressed in yolk sac blood islands, but is expressed in fetal liver, the opposite expression pattern of ɛy globin. In the absence of Sox6, ɛy globin is ectopically expressed in the fetal liver, demonstrating that Sox6 functions in definitive erythropoiesis.

## Results

### Persistent Expression of the Embryonic Globin, ɛy, in Sox6-Deficient Mice

The ɛy globin gene was initially identified as an upregulated transcript in the *p* ^100H^ mouse using subtractive hybridization to identify Sox6 downstream targets. This initial observation was confirmed in an independent knock-out allele of Sox6 [13] using real-time polymerase chain reaction (PCR) (unpublished data). Real-time PCR was used to quantitate the expression levels of other globin genes in *p^100H^* mutant and WT mouse livers at two developmental stages, 15.5 dpc and 18.5 dpc. As shown in Figure 1, the ɛy gene is expressed at high levels at both time points in mutant mice, in contrast to the decline in expression observed in WT mice. No difference was seen between WT and heterozygous mice (unpublished data). Interestingly, the expression levels of the other two embryonic globin genes (ζ and βh1) are also higher in *p^100H^* homozygous mice, compared with WT mice, but to a much lesser extent than seen for ɛy globin at 18.5 dpc (Figure 1). Moreover, the expression level of adult β globin is also somewhat higher in *p^100H^* homozygous mice than in WT mice at 18.5 dpc (Figure 1). Perinatal lethality of mutant mice (presumably from the heart defect [14]) precludes us from evaluating postnatal globin expression. The graphs in Figure 1 illustrate real time PCR results that were performed in triplicate (standard deviation of the data is shown by error bars). Because all of the assays were performed at the same time with the same internal control, the levels shown are relative levels and are thus comparable across all samples and are in agreement with previously published results for WT fetal mice [29]. We note that the level of ɛy expression in the livers of 15.5 dpc and 18.5 dpc homozygous mutant mice is statistically equivalent to the level of βmaj/min expression in the livers of 15.5 dpc and 18.5 dpc homozygous WT mice.

**Figure 1:**
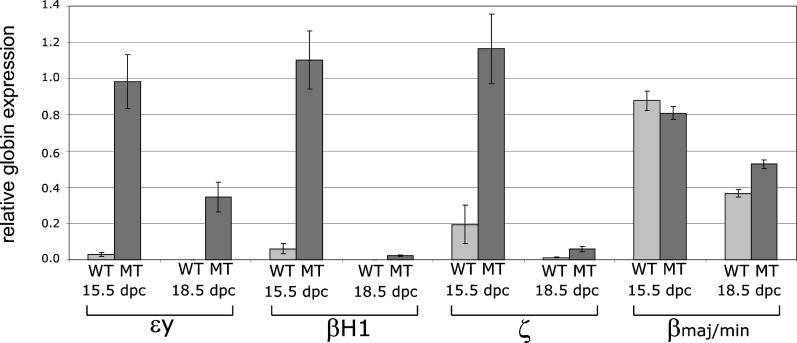
Real-Time PCR of Globin Genes The levels of expression of ɛy, βh1, zeta, and βmaj/min were measured at 15.5 dpc and 18.5 dpc in homozygous WT and *p^100H^* mutant littermates by real-time PCR (see Materials and Methods). Relative expression levels in the livers of each genotype are graphed for each globin gene (performed in triplicate and normalized with GAPDH). Standard deviation is indicated by bars.

### Transfection Studies Using GM979 Cells Indicate That Sox6 Directly Represses the ɛy Gene Promoter at the Transcriptional Level

Real-time PCR assays (Figure 1) measure steady-state levels of ɛy mRNA, not transcriptional activity of the ɛy promoter. To investigate whether Sox6 directly acts on the ɛy gene promoter at the transcriptional level, we used an in vitro transient transfection assay and GM979 cells, a murine erythroleukemic cell line that expresses both ɛy and adult beta globins [30]. We generated an ɛy promoter reporter construct (E-Luc) by fusing a micro-LCR (μLCR) element (2.5 kb) [31] to the ɛy proximal promoter (2.2 kb), followed by the luciferase reporter gene, as shown in Figure 2A (detailed in Materials and Methods). Overexpression of Sox6 in GM979 cells by transient transfection leads to a dosage-dependent repression of E-Luc reporter activity (Figure 2B). In contrast, overexpression of a truncated Sox6 protein that lacks its HMG domain [32] (similar to the *p* ^100H^ mouse allele) fails to repress E-Luc activity (Figure 2B). These data indicate that Sox6 acts to repress the ɛy promoter at the transcriptional level.

**Figure 2:**
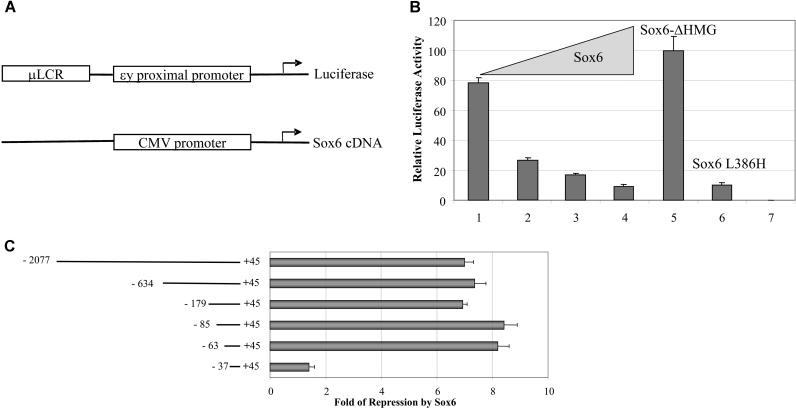
The Effect of Sox6 on the ɛy Promoter (A) Constructs of the ɛy promoter reporter (E-luc) and Sox6 overexpression vector. The E-luc reporter construct consists of a 2.5-kb μLCR element, a 2.2-kb ɛy proximal promoter, and the luciferase reporter in the pGL-3 basic plasmid (see Materials and Methods). Sox6 expression is driven by the CMV promoter. (B) Sox6 represses ɛy promoter activity in a dosage-dependent manner. In GM979 cells, the E-Luc ɛy promoter reporter construct was co-transfected (1) without overexpression of Sox6; (2–4) with increasing amounts of CMV-Sox6 overexpression vector; (5) with a truncated version of Sox6 that lacks its HMG domain; (6) with a mutant version of Sox6 (L386H) that has previously been shown to abolish interaction with CtBP2; or (7) with an empty reporter plasmid (without ɛy promoter and μLCR element). (C) Promoter deletion analyses to delimit the critical sequence. The 2.2-kb proximal promoter or deletions of it, as indicated on the left (numbering relative to +1 = the transcription start site of ɛy globin, see Materials and Methods), were engineered in reporter constructs as in (A) and were transfected along with CMV driven Sox6 to GM979 cells (see Materials and Methods for details). The relative repression by Sox6 on the activity of the different reporter constructs is shown. All experiments were done in triplicate.

Sox6 has been shown to act as a repressor and to interact with a widely expressed co-repressor, CtBP2, on the *fgf-3* promoter [5]. CtBP2 is expressed in GM979 cells (unpublished data). To investigate whether the interaction with CtBP2 is required for Sox6 repression of the ɛy promoter, we introduced a point mutation (L386H) in the Sox6 protein that has been previously reported to be sufficient to abolish Sox6-CtBP2 interaction [5]. This amino acid change is not in the HMG DNA binding domain. However, this mutant version of Sox6 retains the ability to repress the ɛy promoter in the transfection assay (Figure 2B), indicating that Sox6 represses the ɛy promoter in a CtBP2-independent manner. Deletion analysis of the ɛy promoter, as shown in Figure 2C, defined a region (−63 to −37) within the ɛy proximal promoter that is critical for Sox6 repression. Analysis of this short region reveals two Sox/Sox6 consensus binding sites [5] (Figure 3A).

**Figure 3:**
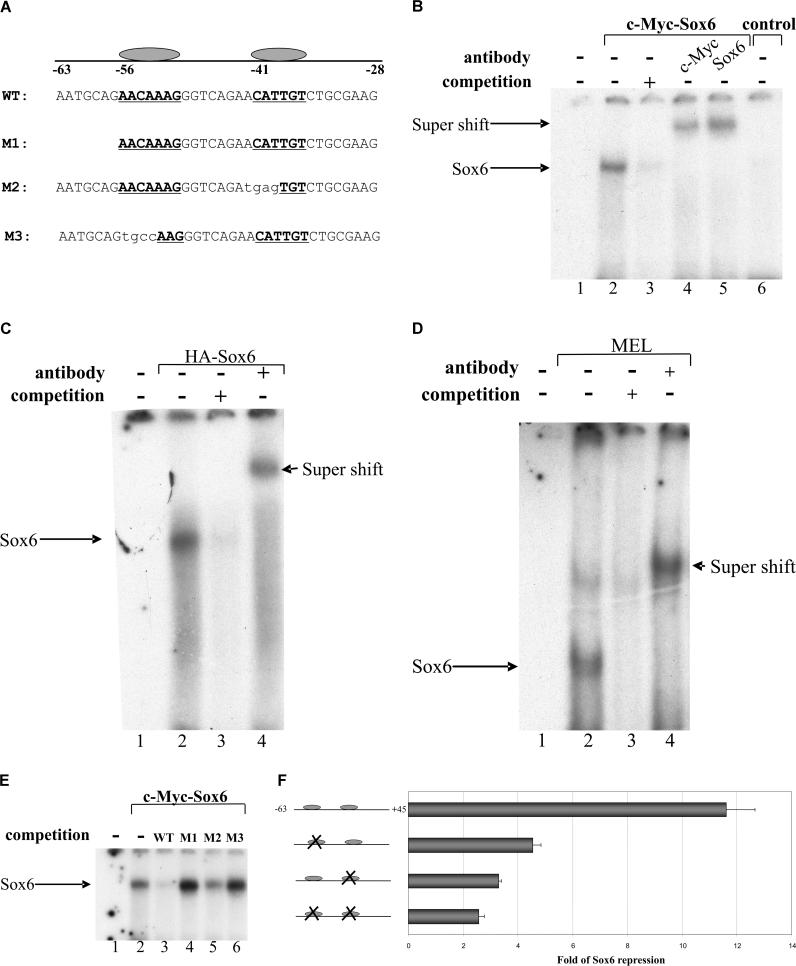
Analysis of the Minimal Region (36 bp) of the Proximal ɛy Promoter Responsive to Sox6 (A) The sequence of the 36-bp fragment and its mutant versions used in EMSA. The WT 36-bp DNA sequence (−63 to −28) of the ɛy globin proximal promoter contains two Sox/Sox6 consensus binding sites, shown in bold underline. Versions with truncation of this sequence (M1) or mutation of one of the two consensus binding sites (M2 and M3) are also shown. (B) EMSA with c-Myc-tagged Sox6. EMSA was performed using the 36-bp radio-labeled WT probe (as shown in (A)) and c-Myc tagged Sox6 translated in vitro using reticulocyte lysate (see Materials and Methods). Lane 1: radio-labeled free probe (run out of the gel); Lane 2: no competition, no antibody; Lane 3: competition with 200-fold excess cold probe, no antibody; Lane 4: no competition, c-Myc antibody (producing a supershift); Lane 5: no competition, Sox6 antibody (producing a supershift); Lane 6: no competion, no antibody using in vitro translated vector containing c-Myc, but not Sox6. (C) EMSA with HA-tagged Sox6. EMSA was performed similarly as in (B) using HA-tagged Sox6 translated in vitro. Lane 1: radio-labeled free probe (run out of the gel); Lane 2: no competition, no antibody; Lane 3: competition with 200-fold excess cold probe, no antibody; Lane 4: no competition, HA antibody (producing a supershift). (D) EMSA using MEL cell nuclear extracts and the 36-bp WT probe. Lane 1: radio-labeled free probe (run out of the gel); Lane 2: no competition, no antibody; Lane 3: competition with 200-fold excess cold probe, no antibody; Lane 4: no competition, Sox6 antibody (producing a supershift). (E) EMSA with c-Myc-tagged Sox6, WT and mutant versions of the 36-bp fragment in competition. EMSA was performed using the radio-labeled 36-bp WT probe and the c-Myc tagged Sox6 translated in vitro*.* Lane 1: radio-labeled free probe; Lane 2: no competition, no antibody. Competition was performed using 200-fold excess cold probes corresponding to WT (Lane 3), M1 (Lane 4), M2 (Lane 5), and M3 (Lane 6). (F) Both consensus Sox/Sox6 binding sites are required for Sox6 responsiveness. GM979 cells were transfected with a reporter construct (Figure 2A) containing −63 to +45 of the ɛy proximal promoter together with the CMV-Sox6 overexpression vector. Mutations of the consensus binding sites were also tested (M3, M2, M2 plus M3, see (A)). The fold repression of Sox6 with the WT or mutant constructs is shown. The baseline activities of the mutagenized reporter constructs are comparable to the WT construct.

### EMSA and ChIP Assays Show that Sox6 Directly Binds to the ɛy Promoter

Sox6 might repress the ɛy promoter, either through direct physical contact with the promoter or by regulating intermediates affecting the ɛy promoter. To investigate whether Sox6 is directly associated with the ɛy promoter, we first performed electrophoretic mobility shift assays (EMSA) using a c-Myc-tagged Sox6 in a reticulocyte lysate-based transcription/translation in vitro system. The probes used are listed in Figure 3A. The 36–base pair (bp) WT probe corresponds to the critical region of the ɛy promoter defined in our promoter deletion analyses. This probe contains two consensus Sox/Sox6 binding sites. Also included in our EMSA are three mutated probes that are, either truncated (M1), or mutated (M2 and M3) in Sox/Sox6 binding sites (Figure 3A). Sox6 is able to physically associate with the 36-bp region (Figure 3B) within the ɛy promoter defined by the deletion analysis experiments (Figure 2C). The 36-bp probe was shifted by the tagged Sox6 protein. Moreover, both c-Myc and Sox6 antibodies supershift the band, indicating that the binding is Sox6-specific. To rule out the possibility that the c-Myc tag itself binds to the probe, an HA-tagged Sox6 was used in another EMSA that confirmed these results (Figure 3C).

Next, nuclear extracts from MEL cells were used in EMSA employing the same 36-bp probe. MEL cells, a murine erythroleukemic cell line, express adult β globins, but not ɛy [33]. Sox6 directly binds to this DNA sequence in MEL cells (Figure 3D). The intact consensus Sox/Sox6 binding sites of the DNA probe are required for the binding, as shown in the competition assay (Figure 3E). Ablation of putative Sox/Sox6 binding sites (M1 and M3) abolish their ability to compete in EMSA (Figure 3E). The M2 mutant probe may compete partially with WT binding.

To investigate the functional significance of the intact Sox/Sox6 binding sites, the ɛy promoter reporter constructs with mutagenized Sox/Sox6 binding sites were co-transfected with the Sox6 overexpression vector into GM979 cells. Consistent with the EMSA results, the mutant ɛy promoter reporter constructs (with either one or both Sox/Sox6 binding sites mutagenized) do not result in significant promoter repression in transfection studies (Figure 3F). Thus, both sites are required for maximal repression of ɛy by Sox6, but not to the same degree.

We also tested whether Sox6 binds to the ɛy promoter in vivo using chromatin immunoprecipitation (ChIP) (Figure 4). The Sox6-containing complex was immunoprecipitated from MEL cells or from liver cells of 15.5 dpc WT mice using Sox6 antibody. Figure 4 shows that the ɛy proximal promoter is readily immunoprecipitated with Sox6 antibody in both MEL cells and liver cells. Normal IgG was used as a negative control (Figure 4A). The above data (Figures 3 and 4) clearly indicate that Sox6 acts as a repressor of the ɛy gene by directly binding to the ɛy promoter, probably as a dimer.

**Figure 4:**
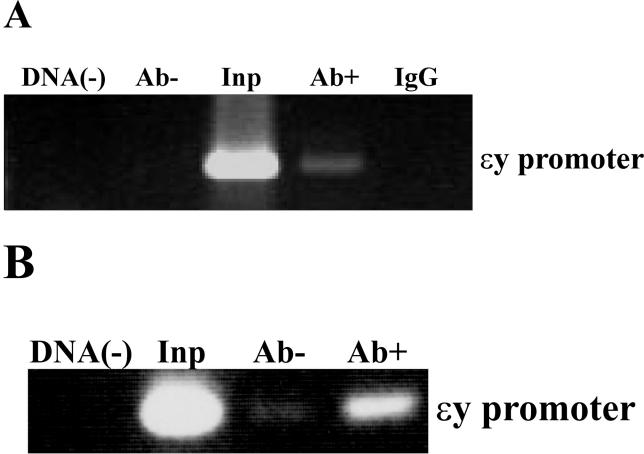
ChIP Assay MEL cells (A) and 15.5-dpc fetal liver cells (B) were treated as detailed in Materials and Methods. 10% of the sample was saved as total input (Inp); remaining samples were divided: plus Sox6 antibody (Ab+), minus Sox6 antibody (Ab−), as well as no DNA (DNA−) and normal rabbit IgG (IgG) that served as negative controls. Other controls for these experiments included PCR within the promoter of the α-globin gene and intron 24 of the *p* gene. Both were negative (unpublished data). PCR was carried out using primer pairs flanking the Sox/Sox6 binding sites (see Material and Methods) of the ɛy proximal promoter. For all reactions, we used 2 μl of immuno-precipitated DNA and 2 μl of 1/100 total input. Semiquantitative PCR was done within the exponential range. Multiple independent experiments were done.

### The Persistent Expression of ɛy Globin in Sox6-Deficient Mice Is Due to a Defect in the ɛy-Gene–Silencing Mechanism in Definitive Erythroid Cells

Normally, the ɛy globin gene is exclusively expressed in primitive erythrocytes and silenced in definitive erythrocytes. To determine whether the persistent expression of ɛy globin is due to residual primitive erythrocytes or is due to ectopic expression of ɛy globin in definitive erythrocytes, we examined the spatial pattern of ɛy globin transcripts in mouse embryos by in situ hybridization (Figure 5). As expected, ɛy globin is not expressed in the WT 14.5-dpc liver, the site of definitive erythropoiesis in the fetus. In contrast, abundant ectopic ɛy mRNA expression is seen in the liver of 14.5-dpc mutants (Figure 5 A–D). However, the expression of βmaj/min globin is equally abundant in both WT and *p^100H^* mutant mice (Figure 5 E and F). These data demonstrate that the persistent high levels of ɛy are due to ectopic expression in the definitive erythroid cells that mature in the fetal liver, suggesting that there is an intrinsic defect of the ɛy silencing mechanism in Sox6-null mice.

**Figure 5:**
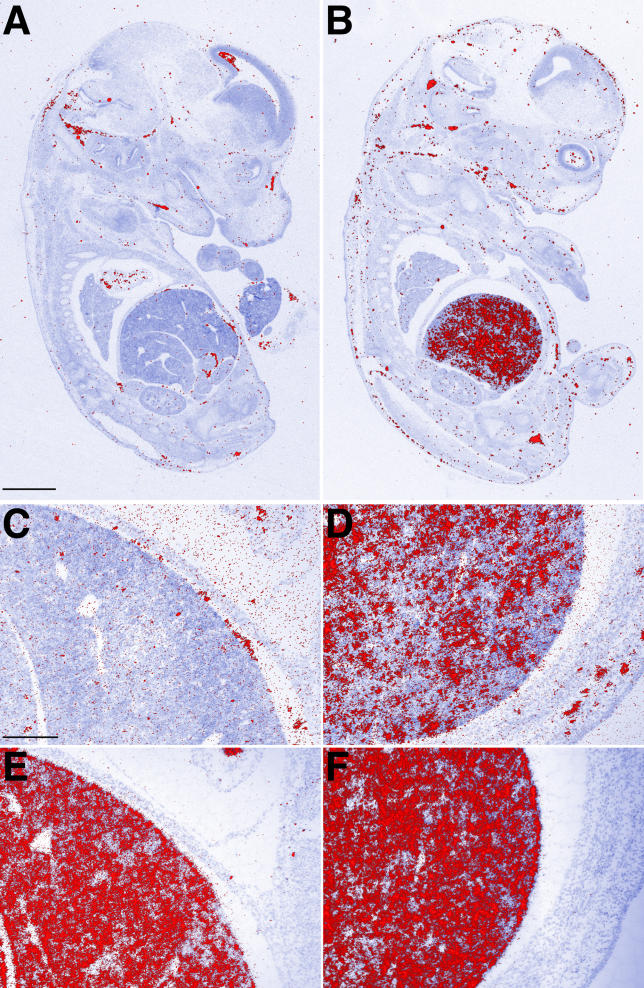
In Situ Hybridization of ɛy and βmaj/min transcripts in WT and *p^100H^* Mutant Mice at 14.5 dpc Expression of ɛy globin (A–D) and β globin (E and F) in sagittal sections of 14.5-dpc fetuses is shown in pseudocolor (red). (A) WT fetus. (B) *p^100H^* Homozygous mutant fetus. (C and D) Inset boxes are shown. Prominent expression of ɛy globin is only detected in the liver of mutant mice. (E and F) In contrast, both WT and mutant mouse livers express adult βmaj/min, respectively. No signals were detected above background using sense probes (unpublished data). The size bars represent 1 mm for (A) and (B); 100 μm for (C–F).

### The Expression Pattern of Sox6 Suggests a Role in Definitive Erythropoiesis

The observation that Sox6-deficient mice ectopically express ɛy globin in liver, where definitive erythroid cells mature, suggests that Sox6 is an important regulator in definitive erythropoiesis. To determine the temporal and spatial expression pattern of Sox6*,* Northern blot and in situ hybridization assays were employed. As shown in Figure 6A, Sox6 is detectable by Northern blot beginning at 10.5 dpc, coincident with the temporal onset of definitive erythropoiesis in the liver. Furthermore, in situ hybridization shows that Sox6 is highly transcribed in 12.5-dpc liver, but not in yolk sac blood islands at 7.5 dpc (Figure 6B). Therefore, Sox6 expression is temporally and spatially coincident with definitive, but not primitive, erythropoiesis. These data, taken together with the observation that ɛy globin is highly expressed in the liver cells of 14.5-dpc *p^100H^* mutant mice (Figure 5), demonstrate that Sox6 functions in definitive erythropoiesis.

**Figure 6:**
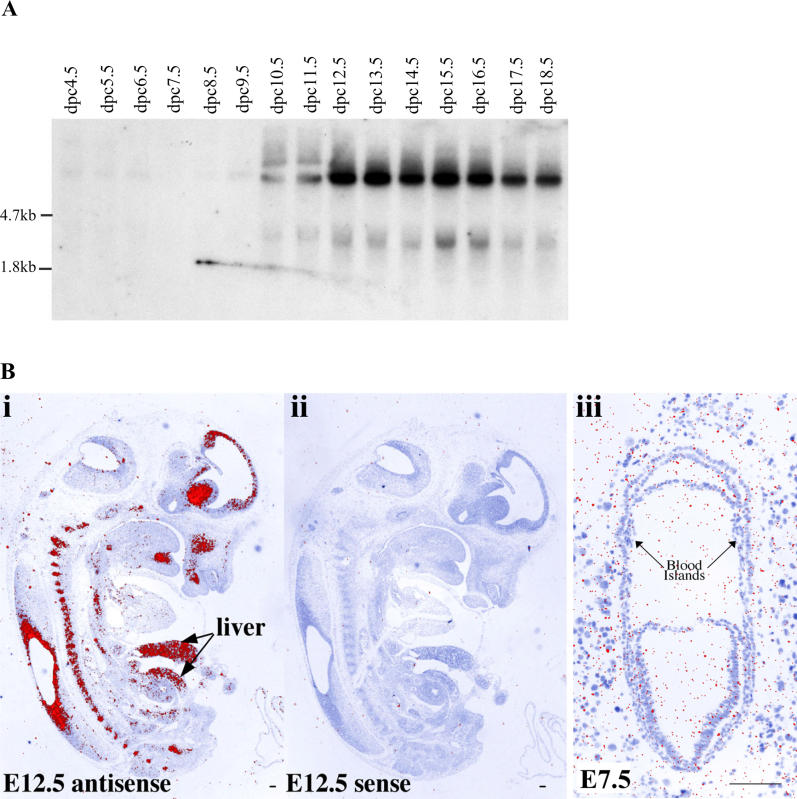
Expression Pattern of Sox6 by Northern Blot and In Situ Assays (A) Sox6 expression during embryonic development shown by Northern blot. Each lane contains 20 μg of total RNA from embryos whose ages are listed above each lane as dpc. The filter was hybridized with a ^32^P-labeled 575-bp mouse Sox6 cDNA fragment (nucleotides 1353–1927). Numbers on the left are sizes of standard marker fragments in kb. (B) Sox6 expression shown by in situ hybridization. Panel i: Sagittal section through an E12.5 mouse embryo using antisense Sox6. mRNA distribution is represented by pseudocolored red signal superimposed on the counterstained specimen. Sox6 transcripts are detected primarily in the fetal liver, developing nervous system, chondrocytes and craniofacial area. Panel ii: The sense control probe shows no signal above background. Panel iii: E7.5 embryo hybridized to antisense probe for Sox6. No signal is detected above background specifically in blood islands (or with the sense probe, unpublished data). The size bars represent 100 μm.

### Mutant *p^100H^* Mice Have Higher Numbers of Nucleated Red Cells

Among the other Sox6 effects in erythropoiesis, we have noticed that there are more nucleated red blood cells circulating in *p^100H^* mutant mice than in WT mice at 14.5 dpc and 18.5 dpc (Figure 7A). However, at postnatal day 10.5, we do not see circulating nucleated red cells in either WT or mutant mice, suggesting that this may be a transient effect. In addition**,** the mutant liver shows a significant increase in hematopoietic precursor cells including nucleated erythrocytes at 18.5 dpc (Figure 7B). This alteration is noted as early as 14.5 dpc. These observations suggest that besides silencing the ɛy globin gene, Sox6 may affect red cell maturation.

**Figure 7:**
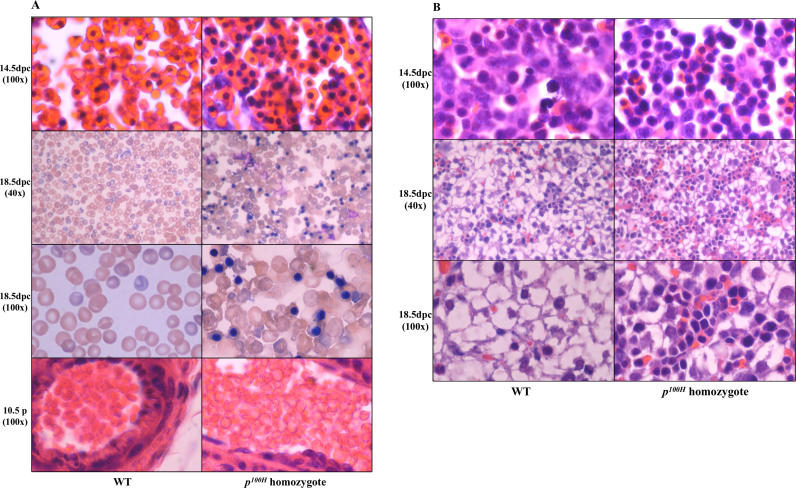
The Blood and Liver Phenotype of WT and *p^100H^* Homozygous Mice (A) Red blood cells of WT mice (left panels) and *p^100H^* homozygous mice (right panels) are shown at the indicated ages. (B) Liver cells of WT mice (left panels) and *p^100H^* homozygous mice (right panels) are shown at the indicated ages.

## Discussion

In this report, we show that Sox6 is a novel factor in the complicated regulation mechanism of globin genes. In the Sox6 null mouse, there is a transient effect on the embryonic globin genes, ζ and βH1, and a persistent upregulation of the ɛy globin gene. Sox6 directly regulates and binds to the proximal promoter of ɛy gene and represses the ɛy-globin gene in definitive erythropoiesis.

Sox6 belongs to group D of the Sox family of proteins that includes Sox5, 12, 13, and 23 [34]. Group D Sox proteins contain a coiled–coiled domain that mediates homo- and heterodimerization [6,35]. Functionally, dimerization of Sox5 and Sox6 has been shown to greatly increase the binding efficiency of the two Sox proteins to DNA that contains adjacent Sox sites [6]. In addition, Sox6 binds more strongly to an HMG-box dimer motif than to a single HMG-box motif [5]. Therefore, it appears that target genes for group D Sox proteins, such as Sox6, probably harbor pairs of HMG binding sites with a configuration compatible with binding of D-Sox protein dimers. Indeed, in the present study, the defined Sox6 target sequence of the ɛy promoter contains two Sox/Sox6 consensus sites (Figure 3A). Functionally, both sites are essential for Sox6 binding to the ɛy promoter and repression of its activity (Figure 3E and 3F). These observations suggest that Sox6 binds to this sequence of the ɛy promoter either as a homodimer or as a heterodimer with other Sox proteins. Because Sox proteins recognize a short 6-bp core-binding sequence that allows for considerable degeneracy, the specificity of their actions is thought to rely upon interactions with other transcription factors [36]. In our EMSAs, we had to run the electrophoresis on a 4%–6% gel for at least 4–8 h to detect the Sox6-associated band, suggesting that Sox6 is part of a high molecular weight complex. A few other ɛy globin repressors have been reported to bind to DNA sequences near the Sox/Sox6 consensus sites, including the DRED complex [37] and COUP-TF [38]. Sox6 might interact with these factors and form a large repression complex. Identification of other components of the Sox6-containing complex associated with the ɛy promoter will shed light on its mechanism of repression.

Sox proteins bind and bend linear DNA by partial intercalation in the minor groove, and can also bind to four-way junctions [2–4]. Therefore, one attractive model to explain how Sox6 proteins control gene expression is that they function as architectural factors bound to DNA, influencing local chromatin structure by bending DNA and by assembling multiprotein transcriptional complexes. By changing the local chromatin structure, Sox6 could either interfere with binding of other activators to the promoter or facilitate binding of other repressors. Another example of a repressor that interferes with an activator on the ɛy promoter is DRED. DRED interferes with EKLF, an activator, in binding to the ɛy promoter [39]. Two HMG architectural proteins (distantly related to the Sox family of transcription factors), HMG-I and HMG-Y, were demonstrated to bind to the human adult β globin silencers (silencers I and II) and cause bending of the DNA, facilitating the binding of other repressors [40].

Sox6 expression is temporally and spatially coincident with definitive (but not primitive) erythropoiesis (Figure 6), and Sox6 represses ɛy globin expression both in vivo (Figure 1) and in vitro (Figure 2). Moreover, in situ hybridization clearly shows that the persistent expression of ɛy globin in *p^100H^* mutant mice is due to defects in the silencing mechanism of definitive erythropoiesis that takes place in the liver (Figure 5). Taken together, these data demonstrate that Sox6 functions in definitive erythropoiesis to silence ɛy globin expression.

The expression level of ɛy globin in homozygous Sox6 null mice at 15.5 dpc and 18.5 dpc is statistically equivalent to the level of βmaj/min expression in the livers of 15.5-dpc and 18.5-dpc homozygous WT mice (Figure 1). This demonstrates that ectopic expression of the ɛy globin gene is quite robust in homozygous mutant mice. The expression levels of two other embryonic globin genes (ζ and βh1) are also higher in *p^100H^* homozygotes, compared with WT. Like ɛy, levels of ζ and βh1 are dramatically higher in mutant mice at 15.5 dpc. However, unlike ɛy globin, ζ and βh1 decline in expression by day 18.5 dpc (Figure 1), suggesting that ɛy is regulated differently than ζ and βh1. It is possible that Sox6 has a general effect on embryonic globin genes (and erythrocyte maturation) in addition to a specific role in silencing ɛy.

Although most *p^100H^* mutant mice die just after being born, a rare few survive longer. None have been observed to live longer than 2 wk after birth [14]. We examined a single archived sample of liver RNA from a mutant mouse on postnatal day 13.5 for globin gene expression and detected high levels of ɛy globin in this RNA sample, compared with undetectable ɛy RNA in WT control mice. At this point in development, the levels of ζ and βh1 RNA were undetectable both in mutant and WT; however, adult β-like globin RNA levels were moderately elevated in the mutant RNA compared with WT (unpublished data), similar to what we observe at 18.5 dpc (Figure 1). These findings suggest that Sox6 continues to function postnatally to silence ɛy globin expression and has a unique function in the regulation of ɛy-globin. The mechanism by which Sox6 regulates the other embryonic globin genes remains to be elucidated.

Sox6 has other effects in erythropoiesis, including a delay in enucleation/maturation in *p^100H^* mutant mice. This may be the result of indirect effects, such as stress-induced proliferation (resulting from cardiac defects) and/or anemia. Severe anemia can lead to rapid premature release of red cells, prior to their complete maturation. However, the hematocrit of 18.5-dpc mutant mice is only 20% lower than that of WT (unpublished data), and this mild anemia is probably not sufficient to explain the extent of nucleated red cells. Alternatively, Sox6 itself may play a role in red cell terminal differentiation, as it has been shown to be an important factor in cardiac [15], neuronal [10], astrocytic [11], and cartilage differentiation programs [32,41–44].

The restoration of normal enucleation of red cells in Sox6-deficient mouse by postnatal day 10.5 may result from functional compensation of other Sox proteins (expressed at later developmental stages), since functional redundancy is a recurring theme with Sox proteins [13,45,46]. Moreover, erythropoiesis has already shifted from fetal liver to bone marrow by postnatal day 10.5. The accompanying change in the microenvironment of red cell production may permit normal enucleation. Identification of Sox6 downstream target genes and its interacting proteins will shed light on the role of Sox6 in red cell terminal differentiation and the enucleation process.

Recently, in vivo and in vitro analyses suggest that reactivation of human ɛ-globin would be therapeutically beneficial to adults with sickle cell disease [47], providing a rationale for detailed investigations into the molecular basis of ɛ-globin gene silencing. The present study identifies a novel repressor, Sox6, which binds to the ɛy proximal promoter, potentially as part of a larger repression complex. Because murine Sox6 and its human counterpart are 94% identical at the amino acid level [48], it is possible that human Sox6 may also be important in human ɛ globin silencing. There is significant sequence homology between the human and mouse ɛ promoter regions, and the human promoter contains at least two potential Sox6 binding sites. Indeed, the existence of a silencer of the human ɛ globin gene has been proposed [49,50]. Thus, elucidation of the Sox6 repression mechanism and identification of other components of the Sox6-containing complex may further our understanding of ɛ globin regulation and potentially reveal additional molecular targets for the treatment of sickle cell anemia and β thalassemias.

## Materials and Methods

### Plasmid construction.

The ɛy promoter deletion reporter plasmid (E-luc) was generated by PCR amplification of the ɛy proximal promoter. A 2.2-kb fragment upstream of the ɛy globin initiation codon (ATG) was used, because it has been shown that all sequences required for ɛ gene silencing are located within a 3.7-kb EcoRI fragment containing about 2 kb of sequence upstream of the ɛ globin gene cap site [50]. Nucleotide numbering is relative to the transcription start site. The transcriptional start site is based on the longest cDNA in the Fantom (Functional Annotation of Mouse) database. The PCR primers contained XhoI and HindIII sites that were used to clone the ɛy promoter fragment upstream of the firefly luciferase gene in pGL3 Basic (Promega, Madison, Wisconsin, United States). A 2.5-kb SstI/XhoI fragment of μLCRβ 9.3 (micro LCR; [31]) was then inserted upstream of the ɛy promoter in the above pGL3 Basic plasmid, resulting in a reporter construct in which luciferase expression is driven by the ɛy promoter. A series of deletion constructs of the ɛy promoter were generated similarly. Forward primers with an XhoI site include: MHB1457, 5′CCGCTCGAGTGCTAGGCAAACACTCA3′ (−2077 to −2052); MHB1503, 5′CCGCTCGAGTCTCTACACTGTCACTCCCTG3′ (−634 to −605); MHB1505, 5′CCGCTCGAGGGAGCCAAAAAAAGAATGC3′ (−197 to −169); MHB1506, 5′CCGCTCGAGCTGACCAATGGCTTCAAAG3′ (−85 to −58); MHB1532, 5′CCGCTCGAGAATGCAGAACAAAGGGTCAGA3′ (−63 to −34); and MHB1507, 5′CCGCTCGAGGTCTGCGAAGAATAAAAGGC 3′ (−37 to −9). All forward primers were used in combination with the reverse primer HindIII site: MHB1477, 5′CGGAAGCTTGGGAGGTTGCTGGTGA3′ (+45 to +20).

Sox6-pcDNA3.1 [15] was used to overexpress Sox6. A truncated version of the Sox6 overexpression construct (Sox6-ΔHMG-pcDNA3.1) that lacks the HMG domain was generated, as described by others [32]. Mutagenesis of Sox/Sox6 consensus binding sites of the ɛy promoter were done by PCR. Forward primers used to generate these mutagenized ɛy promoter reporter constructs include: MHB1661, 5′CCGCTCGAGAATGCAGTGCCAAGGGTCAGAACATTGTCTGCGAAG3′ (−63 to −19); MHB1662, 5′CCGCTCGAGAATGCAGAACAAAGGGTCAGATGAGTGTCTGCGAAGAA3′ (−63 to −16); and MHB1663, 5′CCGCTCGAGAATGCATGCCAAGGGTCAGATGAGTGTCTGCGAAGAA 3′ (−63 to −18).

### Quantitation of globin mRNA.

RNA was first reverse transcribed to cDNA. Primers for cDNA PCR amplification of globin genes were obtained from Primerbank [51]. All primers were searched against the NCBI database to confirm specificity. For ɛy globin: MHB1666, 5′TGGCCTGTGGAGTAAGGTCAA3′; and MHB1667, 5′GAAGCAGAGGACAAGTTCCCA3′. For ζ globin: MHB1668, 5′CTACCCCCAGACGAAGACCTA3′; and MHB1669, 5′CTTAACCGCATCCCCTACGG3′. For βH1 globin: MHB1672, 5′TGGACAACCTCAAGGAGACC3′; and MHB1673, 5′ACCTCTGGGGTGAATTCCTT3′. For βmaj/min globin: MHB1674: 5′ATGGCCTGAATCACTTGGAC3′; and MHB1675, 5′ACGATCATATTGCCCAGGAG3′. Using the SYBR green supermix kit with ROX (Bio-Rad, Hercules, California, United States), PCR amplification was run on an ABI7000 (Applied Biosystems, Foster City, California, United States) at the University of Arizona core facility. All PCR was performed in a 25-μl reaction with 12.5 μl SYBR green supermix. GAPDH mRNA levels were used as control for input RNA. Standard curve analyses were performed to test the efficiency of the amplifications. Triplicates were done for each PCR reaction. Relative quantitative values were calculated in the ABI Prism 7000 SDS Software (Applied Biosystems) and normalized to GAPDH in Microsoft Excel (Redmond, Washington, United States).

### In situ hybridization.

Antisense probes were designed to murine ɛy globin nucleotides 509–584; βmaj globin nucleotides 458–549; and mouse Sox6 nucleotides 1353–1927. Embryos were fixed overnight by immersion in 4% paraformaldehyde, embedded in paraffin, sectioned at 5 μm, and adhered to charge modified slides (VWR, West Chester, Pennsylvania, United States). Slides were processed for in situ hybridization as described [52] using in vitro transcribed RNA probes labeled with ^33^P. Darkfield and brightfield images were obtained with a Nikon Optiphot microscope (Nikon, Melville, New York, United States) and SPOT RT-Slider digital camera (Diagnostic Instruments, Sterling Heights, Michigan, United States). Objectives used were 1× (NA = 0.04) and 10× (NA = 0.5). Images were processed, pseudocolored, and combined using Photoshop (Adobe, San Jose, California, United States) software with Fovea Pro (Reindeer Graphics, Asheville, North Carolina, United States) plugins. Original images are available.

### Histology.

18.5-dpc embryos were exsanguinated and peripheral blood smears were prepared from both mutant and WT mice. The slides were Wright-stained and read by DAF. For whole mount analysis, 14.5-dpc WT and mutant embryos, and postnatal day–10.5 mice were fixed in 10% formalin, paraffin-embedded, sectioned at 5 μm, and stained with hematoxylin and eosin. Liver samples (at 14.5 dpc and 18.5 dpc) were prepared in a similar manner. Images were obtained with Nikon Labophot-2 microscope. Objectives used were E Plan 40/0.65 160/0.17 Nikon (40× objective), E Plan 100/1.25 oil 160/0.17 Nikon (100× objective). The camera was a Nikon Coolpix 4300. Original images are available.

### Northern blot.

A mouse embryonic tissue Northern blot filter (Seegene, Rockville, Maryland, United States) was hybridized with a Sox6 probe generated by RT-PCR (nucleotides 1353–1927) and labeled with [α-^32^P]dCTP, by random primer labeling (RediprimeII; Amersham Biosciences, Buckinghamshire, England, United Kingdom). The hybridization was performed in phosphate buffered 7% SDS hybridization solution. Blots were washed with 0.2× SSC, 1% SDS at 60 °C prior to exposure to X-ray film (Kodak, Rochester, New York, United States) at −80 °C for 6 d.

### Cell culture and transfection.

GM979 cells (Coriell Cell Repositories, Camden, New Jersey, United States) were cultured in Ham's F12 with 2 mM L-glutamine supplemented with heat-inactivated 10% fetal calf serum (Ivitrogen, Carlsbad, California, United States), penicillin (100 units/ml), streptomycin 100 μg/ml), and L-glutamine (2 mM). MEL cells were cultured in DMEM supplemented as above (without heat inactivating the serum). GM979 cells (4 × 10^5^) in log phase of growth were transfected with plasmids by FuGENE6 (Roche, Indianapolis, Indiana, United States). Cells were transfected with ɛy promoter reporter constructs (500 ng) along with either empty vector or Sox6 overexpression vector (1000 ng). In assays of dosage effect, we used 200 ng, 500 ng, and 1000 ng). pRL-CMV 15ng (Promega) was used as a control for transfection efficiency.

### Nuclear protein extract and in vitro translation of Sox6.

Nuclear extracts were prepared from MEL cells (2 × 10^7^) using a kit (Active Motif, Carlsbad, California, United States). The Sox6 in vitro translation expression vector, tagged with c-Myc and HA, was described before [15]. The translation was performed in a reticulocyte lysate based in vitro translational system (TNT^®^ Quick Coupled Transcription/Translation Systems, Promega). A vector without the Sox6 coding sequence was also translated as a negative control.

### Antibodies.

Sox6 antibodies used in this study were either kindly provided by Dr. Enzo Lalli (Université Louis Pasteur, France [7]) or commercially obtained (Catalog No. sc-17332 X, Santa Cruz Biotechnology, Santa Cruz, California, United States). All Sox6 antibodies generated similar results. c-Myc antibody was purchased from Invitrogen. Normal rabbit IgG antibody was obtained from Upstate Biotechnology (Lake Placid, New York, United States).

### EMSA.

Single-stranded complementary oligonucleotides were annealed and end-labeled with [γ-^32^P] ATP with T4 polynucleotide kinase. EMSA was performed with 5 μg of nuclear proteins from MEL cells or 3 μl of in vitro-translated Sox6 along with the reticulocyte lysate in binding buffer: 100 mM NaCl, 10% glycerol, 200 ng/μl BSA, 50 ng/μl poly (dI-dC) or poly (dG-dC), 10 mM HEPES (pH 7), 0.1 mM EDTA, 0.25 mM DTT, 0.6 mM MgCl_2_. For competition or supershift assays, the indicated unlabelled oligonucleotide competitor (200-fold molar excess) or antibody (3 μl) was added 30 min to 60 min prior to addition of radiolabeled probe. Following addition of the radiolabeled probe, the samples were incubated for 30 min or 60 min at room temperature and loaded on a 4% or 6% (w/v) polyacrylamide gel. Electrophoresis was performed at a constant 19 mAmp for 4–8 h at room temperature, and the gels were dried prior to autoradiography. Antibodies used for supershift analyses included c-Myc, HA, and Sox6 antibodies (described as above). The DNA sequences of the oligonucleotides are as follows (only forward oligos are listed): For the 36-bp WT probe: 5′AATGCAGAACAAAGGGTCAGAACATTGTCTGCGAAG3′ (MHB1556); for mutant probe 1 (M1): 5′AACAAAGGGTCAGAACATTGTCTGCGAAG3′ (MHB1644); for mutant probe 2 (M2): 5′AATGCAGAACAAAGGGTCAGAtgagTGTCTGCGAAG3′ (MHB1648); for mutant probe 3 (M3): 5′AATGCAGtgccAAGGGTCAGAACATTGTCTGCGAAG3′ (MHB1650).

### ChIP assay.

As described by Nouzova [53], in brief: Cells from MEL cells (4 × 10^7^) or fetal liver cells from three 15.5-dpc WT mice were treated with 1% formaldehyde for 10 min at 37 °C, rinsed in ice-cold 1× Hanks' balanced salt solution with 0.1% EDTA containing protease inhibitors, collected by centrifugation at 4 °C, resuspended in a SDS lysis buffer containing protease inhibitors, and incubated on ice for 10 min. DNA-protein complexes were sonicated to 200 and 600 bp. One-tenth of the sample was set aside for input control, and the remaining sample was precleared with protein A-Sepharose (Amersham Biosciences, Piscataway, New Jersey, United States). Following preclearing, the samples were split into thirds: one sample treated with anti-Sox6, a second treated with normal rabbit IgG, and the third sample without Ab. The last two were used as negative controls. The chromatin-antibody complexes were eluted, and the DNA protein cross-links were reversed with 5 M NaCl at 65 °C for 4 h. Input DNA or immunoprecipitated DNA was used as a template in the PCR reaction. PCR amplification of the ɛy promoter was performed and yielded a 172-bp amplicon, corresponding to nucleotides −31 to** +**140 of the ɛy promoter (primers MHB1688, 5′CGAAGAATAAAAGGCCACCA3′; and MHB1689, 5′GCTTCACCACCAACCTCTTC3′). PCR was performed under the following conditions: 95 ^°^C for 15 min followed by 30 cycles at 95 ^°^C for 30 s, 60 ^°^C for 30 s, and 72 ^°^C for 45 s, ending with a final extension at 72 ^°^C for 5 min.

## Supporting Information

### Accession Numbers

Accession numbers for the genes and gene products discussed in this paper are βH1 globin (GenBank NM_008219), βmaj globin (J00413) (in situ), βmaj/min globin (NM_008220) (real time PCR), ɛy globin cDNA (NM_008221) from the Fantom (Functional Annotation of Mouse) database (http://fantom2.gsc.riken.jp), ζ globin (GenBank NM_010405), and mouse Sox6 (U32614).

## Abbreviations

bp: base pair
ChIP: chromatin immunoprecipitation
dpc: days post coitus
EMSA: electrophoretic mobility shift assay
HMG: high mobility group
PCR: polymerase chain reaction
Sox6: Sry type HMG box
WT: wild-type
